# “CLADE-FINDER”: *Candida auris* Lineage Analysis Determination by Fourier Transform Infrared Spectroscopy and Artificial Neural Networks

**DOI:** 10.3390/microorganisms12112153

**Published:** 2024-10-26

**Authors:** Carlotta Magrì, Elena De Carolis, Vittorio Ivagnes, Vincenzo Di Pilato, Bram Spruijtenburg, Anna Marchese, Eelco F. J. Meijer, Anuradha Chowdhary, Maurizio Sanguinetti

**Affiliations:** 1 Dipartimento di Scienze di Laboratorio ed Ematologiche, Fondazione Policlinico Universitario “A. Gemelli” IRCCS, 00168 Rome, Italymaurizio.sanguinetti@unicatt.it (M.S.); 2 Department of Surgical Sciences and Integrated Diagnostics (DISC), University of Genoa, 16132 Genoa, Italy; vincenzo.dipilato@unige.it (V.D.P.); anna.marchese@unige.it (A.M.); 3 Microbiology Unit, IRCCS Ospedale Policlinico San Martino, 16132 Genoa, Italy; 4 Radboudumc-CWZ Center of Expertise for Mycology, 6500 Nijmegen, The Netherlandseelco.meijer@cwz.nl (E.F.J.M.); 5 Canisius-Wilhelmina Hospital (CWZ)/Dicoon, 6532 Nijmegen, The Netherlands; 6 Medical Mycology Unit, Department of Microbiology, Vallabhbhai Patel Chest Institute, University of Delhi, Delhi 110007, India

**Keywords:** *C. auris*, clades, FTIR, CLADE-FINDER, outbreak management

## Abstract

In 2019, *Candida auris* became the first fungal pathogen included in the list of the urgent antimicrobial threats by the Centers for Disease Control (CDC). Short tandem repeat (STR) analysis and whole-genome sequencing (WGS) are considered the gold standard, and can be complemented by other molecular methods, for the genomic surveillance and clade classification of this multidrug-resistant yeast. However, these methods can be expensive and require time and expertise that are not always available. The long turnaround time is especially not compatible with the speed needed to manage clonal transmission in healthcare settings. Fourier transform infrared (FTIR) spectroscopy, a biochemical fingerprint approach, has been applied in this study to a set of 74 *C. auris* isolates belonging to the five clades of *C. auris* (I-V) in combination with an artificial neural network (ANN) algorithm to create and validate “CLADE-FINDER”, a tool for *C. auris* clade determination. The CLADE-FINDER classifier allowed us to discriminate the four primary *C. auris* clades (I-IV) with a correct classification for 96% of the samples in the validation set. This newly developed genotyping scheme can be reasonably applied for the effective epidemiological monitoring and management of *C. auris* cases in real time.

## 1. Introduction

*Candida auris* is an emerging multidrug-resistant fungal pathogen that has rapidly become a significant and global public health concern. Since its initial identification in Japan in 2009, *C. auris* has been reported in over 60 countries across six continents, causing numerous outbreaks in healthcare settings [1,2]. This pathogen poses a unique challenge due to its ability to colonize the skin, persist in the environment, and quickly develop resistance to multiple classes of antifungal agents, leading to high morbidity and mortality rates among patients [3,4,5]. The increasing prevalence of *C. auris* infections underscores the urgent need for effective strategies to detect, manage, and control this challenging pathogen.

*C. auris* is characterized by a high genetic diversity between its clades, with distinct phylogenetic clades that exhibit geographical clustering and varying antifungal resistance profiles. To date, six main clades have been recognized: South Asian (clade I), East Asian (clade II), African (clade III), South American (clade IV), Iran (clade V) [6,7], and the most recently discovered Indomalayan (clade VI) [8]. Each clade has unique genetic and phenotypic characteristics, which are crucial for understanding the pathogen’s epidemiology and developing targeted antifungal treatment strategies [9]. The accurate determination of these clades is essential for tracking the spread of *C. auris* and implementing appropriate infection control measures.

Common methods for clade classification, such as whole-genome sequencing (WGS) with appropriate phylogenetic analyses, provide high-resolution insights into the genetic relationships among *C. auris* isolates [3]. Clade-typing either relies on WGS or other molecular methods such as multilocus sequence typing or the amplification of conserved clade-specific sequences [10]. However, these methods are time-consuming, costly, and require specialized equipment and expertise, making them less accessible in resource-limited settings [11]. To address these challenges, there is a growing interest in developing computational models that can predict clades based on more readily available genomic or phenotypic data. Fourier-transform infrared (FTIR) spectroscopy is a tool recently introduced in the microbial landscape, especially for outbreak investigations, as it is able to produce an infrared spectrum with a specific absorbance profile for a given strain. While widely used for bacteria, its application in fungal species is limited and it has never been applied in determining *C. auris* clades [12].

Machine learning, a subset of artificial intelligence, offers powerful tools for analyzing complex biological data and making accurate predictions. Recent advancements in machine learning algorithms have demonstrated their potential in various applications, including the classification of genetic sequences, prediction of antimicrobial resistance, and identification of pathogen characteristics [13,14]. By leveraging large-scale genomic datasets, machine learning models can be trained to recognize patterns associated with different *C. auris* clades, enabling rapid and cost-effective clade determination.

Several studies have explored the use of machine learning for predicting antifungal resistance and other phenotypic traits in *C. auris*. For instance, supervised learning techniques, such as decision trees, support vector machines, and neural networks, have been employed to classify genetic sequences and predict pathogen characteristics with high precision [14,15]. These approaches have shown promise in enhancing the accuracy and efficiency of clade prediction models, paving the way for their integration into routine clinical and epidemiological workflows.

The development of a robust prediction model for the clade determination of *C. auris* provides significant benefits for public health. The rapid and accurate classification of *C. auris* clades can facilitate timely infection control measures and guide antifungal therapy, ultimately reducing the burden of *C. auris* infections [16]. Furthermore, clade-specific data can enhance our understanding of the pathogen’s transmission dynamics, resistance mechanisms, and evolutionary history, contributing to more effective strategies for combating this emerging threat.

In this paper, we present a novel prediction model for the clade determination for *C. auris*, leveraging advanced machine learning techniques and FTIR spectroscopy. Our model aims to provide a rapid, accurate, and cost-effective tool for identifying *C. auris* clades, thereby enhancing our ability to monitor and control this challenging pathogen. By integrating recent developments in bioinformatics, pathogen genomics, and FTIR-based spectral analysis, this study contributes to the growing body of knowledge aimed at mitigating the public health threat posed by *C. auris*. We detail the methodologies employed, including data collection, FTIR spectral acquisition, model training, and validation, and discuss the implications of our findings for public health and future research directions.

## 2. Methods

### 2.1. Isolates Collection

A total of 63 molecularly characterized *C. auris* isolates were analyzed in this study, as representatives of the four major distinct clades (I–IV), and their clade was determined as described earlier [6]. Fondazione Policlinico Gemelli, IRCCS, in Rome, investigates *C. auris* outbreaks and is the Italian reference center for *C. auris* surveillance. Forty samples of Clade I were collected from a large nosocomial outbreak identified in northern Italy [17], providing a substantial dataset for this study. The remaining isolates were provided by external research groups, contributing additional diversity to our dataset. Specifically, this included 3 isolates of clade I (India and Pakistan), 6 isolates of clade II (South Korea and Japan), 7 isolates of clade III (India, South Africa and Spain), 11 isolates of clade IV (Colombia, South Africa, Venezuela), and 3 isolates of clade V (Iran). All *C. auris* isolates were retrieved from blood samples. Each isolate was grown at 37 °C for 24 h on Saboraud dextrose agar (SDA) plates (Kima, Padua, Italy) and prepared for the analysis following the standardized protocols reported below to ensure consistency in sample handling and data quality.

### 2.2. Infrared Spectroscopy and Machine Learning Analysis

FTIR analysis was conducted on isolates that had been grown for 24 h at 37 °C on SDA plates. Following the IR Biotyper (Bruker Daltonics, GmbH, Bremen, Germany) manufacturer’s guidelines, *C. auris* yeast cells were suspended in a 70% ethanol solution. The suspension was homogenized by vortexing twice, once before and once after adding 50 µL of deionized water, in 1.5 mL microcentrifuge tubes containing metal beads. For technical replicates, 15 µL of this homogenized suspension was placed in quintuplicate on a silicon sample plate and dried before analysis. To address inter-run variability, three independent experiments (biological replicates) were performed. Each run included quality controls with two infrared test standards in duplicate. The spectra were analyzed in the carbohydrate region (1300–800 cm^−1^) using the IR Biotyper system with its default settings, following previously established quality control criteria [12].

The proposed model combined FTIR with an artificial neural network (ANN) algorithm enhanced by principal component analysis (PCA) to classify *C. auris* isolates into their respective clades. A total of 178 spectra from 16 isolates selected in order to cover all four major clades were analyzed as FTIR training profiles: clade I (50 spectra), clade II (34 spectra), clade III (49 spectra), and clade IV (45 spectra). Due to the limited number of clade V isolates, they were not included in the model but instead utilized as outliers. At first, a bidimensional scatter plot was generated including the 178 infrared spectral profiles representative of all four *C. auris* clades included in the model. A Linear Discriminant Analysis (LDA) was applied for dimensionality reduction; the variance content of each principal component and the cumulative variance was assessed in order to verify the clusters separation of the four *C. auris* spectral profiles. Afterwards, a new classifier was created using the default splicing method, an ANN with the previous PCA as an algorithm, 300 training cycles, and the auto-calculated gamma value. After all parameters were set, the training spectra were used to train the classifier. A cross-validation analysis was generated by a confusion matrix. In the matrix, the predicted clades (rows) were compared against the actual clades (columns), and the model’s accuracy and precision for each clade was calculated.

Subsequently, a set of 273 validation spectra belonging to a total of 58 isolates across the four primary clades (clades I–IV) and the fifth clade (as an outlier) was applied to validate the classifier. A table was generated to easily summarize the validation results obtained and a 2D scatter plot was generated to better visualize the discrimination of the different *C. auris* clades. 

After the acquired spectra were analyzed, the classification results were reported according to the classifiers and to the strain typing method settings applied; the classification results were displayed along with the iso-score. The iso-score summarizes all the scores of the average spectra. The class result with the highest cumulated score wins. Iso-score ranges between 0 and 100 (the higher the score, the closer the sample is in relation to the training set). Iso-score values above 80 indicate that the sample most probably belong to the class in question (in green); averages reaching at least 20 points are reported in yellow. Spectra averaging below 20 specify outliers (in red), meaning that the sample probably does not belong to the class in question at all.

## 3. Results

### 3.1. Classification Model Training

**Figure 1:**
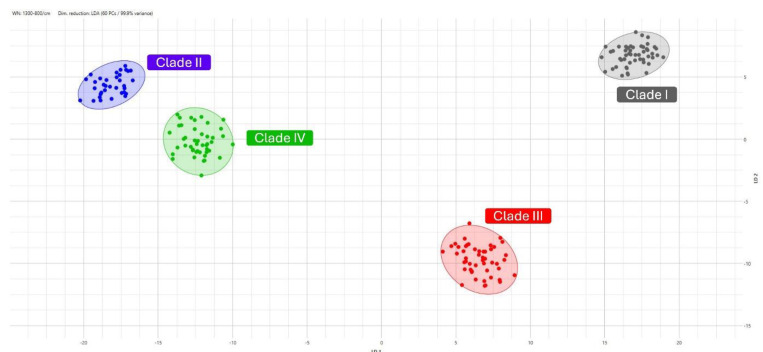
A 2D scatter plot based on the training spectra included in the model. Each point on the plot corresponds to an individual spectrum, colored and grouped by clade: clade I (gray), clade II (blue), clade III (red), and clade IV (green).

Regarding the set of 178 FTIR spectra acquired from the 16 isolates used to build the model, the LDA applied to the *C. auris* infrared profiles allowed us to capture 99% of the variance with 23 principal components (PCs). Based on the FTIR spectra in the scatter plot shown in Figure 1, the clear separation of the clusters, with no overlap, indicates the effectiveness of the LDA in distinguishing between the four distinct clades (I-IV) with a high classification accuracy.

The resulting clade identification outcome is visualized as a confusion matrix in Table 1, illustrating the performance of the clade classification model and reporting the results of the cross-validation for *C. auris* using the IR Biotyper system.

The confusion matrix demonstrated that the classification model correctly allocated isolates across all four clades of *C. auris*. There were no misclassifications, as indicated by the absence of any non-zero entries outside of the diagonal.

### 3.2. Classification Model Validation

Following the results of the run including a total of 273 average spectra from 58 *C. auris* isolates, a classification was performed for the algorithm validation set used for the analysis. In summary, clade I comprised 208 averaged spectra from 46 isolates, with most isolates correctly classified with high reliability; the majority of these isolates showed consistent scores close to 100.0, indicating a strong model performance for this clade. There were a few instances where secondary classifications indicated potential overlap with clade III, but the primary classification was correct. Clade II included 11 average spectra from 3 isolates, and the isolates were generally classified correctly, scoring around 100.0, although an occasional overlap with clade IV was observed. Clade III, represented by 12 average spectra from 3 isolates, presented more challenges. Some isolates were misclassified into clade IV or clade I, and while many achieved high reliability scores, the presence of secondary classifications indicated potential spectral similarities that need further attention. Clade IV, consisting of 18 average spectra from 6 isolates, showed a high classification accuracy without any misclassification. The reliability scores for clade IV were consistently high, confirming the model’s effectiveness in distinguishing this clade from others.

Only two misclassified clades were observed, which are in red, belonging to spectra averages related to samples B11098 and 08-11-93; the first was classified as clade IV and the second as clade IV, instead of I and III, respectively. Based on these results, and excluding the three clade V isolates and those for which a red iso-score was reported, the classifier was able to correctly classify the majority of isolates, 48 out of 50, with a correct clade classification for 96% of the samples.

Clade V spectra, consisting of 24 average spectra from 3 isolates, were used as outliers. They were classified as clade IV or II, evidencing the low ability of the system to discover new clades, as they were not included in the model training.

Of note, as reported in Table 2, the second-best result of the clade I average spectra classification was clade III, highlighting the ability of the algorithm to classify genetically related clades.

**Figure 2:**
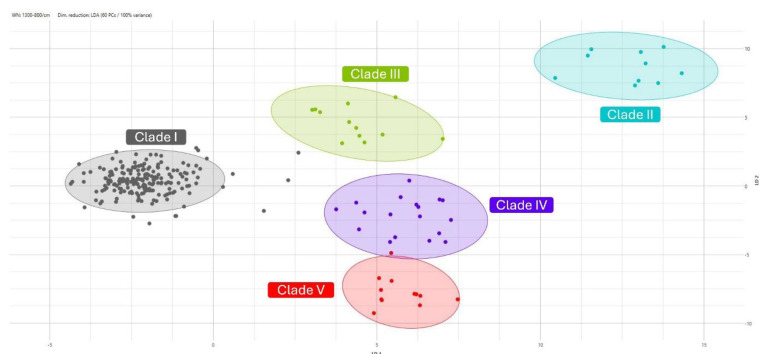
Classification results for FTIR profiles of the validation set of *C. auris* isolates, visualized as a scatter plot, with LDA used for dimensionality reduction. Isolates are colored and grouped by clade: clade I (gray), clade II (turquoise), clade III (green), clade IV (violet), and clade V (red).

Additionally, the scatter plot (Figure 2) visualizes the classification results of *C. auris* isolates into five distinct clades (I, II, III, IV, and V) using LDA for dimensionality reduction. 

The plot captures 99% of the variance using 14 principal components. Clade I, represented by gray dots, shows a tightly clustered group that is well separated from the other clades, indicating its high classification accuracy and distinct spectral characteristics. Clade II, represented by turquoise dots, forms a separate group, suggesting that the classification model is effective. Clade III, represented by green dots, forms a distinct cluster, just as clade IV (violet dots) does. Concerning Clade V (red dots), there is one average profile overlapping with clade IV, while substantial differences in its FTIR spectra still allow us to obtain a separate cluster.

## 4. Discussion and Conclusions

Here, the “CLADE-FINDER” tool was constructed and validated for *C. auris* Lineage Analysis and DEtermination by FTIR spectroscopy and using artificial neural networks. The prediction model for *C. auris* has demonstrated its potential as a powerful tool that can generally accurately differentiate the four clades of this emerging multidrug-resistant pathogen. The model, which integrates advanced machine learning techniques with FTIR spectroscopy, achieved high accuracy in distinguishing between the four primary clades (I, II, III, and IV). The results indicate that this approach can significantly enhance our ability to monitor and control *C. auris* infections, especially in resource-limited settings where reference methods such as WGS or molecular tools may be unavailable [18]. Of note, for identifying clonal outbreaks, short tandem repeat genotyping and/or WGS single-nucleotide polymorphism (SNP) analyses are needed for confirmation [19].

In the training group, the model showed excellent accuracy, precision, and recall across all clades, as evidenced by the confusion matrix, which had no misclassifications. This demonstrates the robustness of the model during its initial training phase. The scatter plot using LDA further confirmed the model’s effectiveness, showing clear separation between the clades with minimal overlap, capturing 95.7% of the variance with nine principal components.

For the validation group, the model maintained a high level of performance, correctly classifying the majority of the isolates with high reliability. Clade I was well separated from the others, indicating the model’s high classification accuracy and the clade’s distinct spectral characteristics. With regard to the remaining clades, no overlaps were observed between clade III and clade IV and between clade II and clade IV, while only one average profile belonging to clade V showed overlap with clade IV, suggesting that there are areas for further model refinement once more isolates from this clade are available [20].

It is noteworthy that the majority of the *C. auris* isolates exhibiting an incorrect clade prediction belonged to a clade other than clade I. As reported in a study by Jamalian et al. [21], a higher proportion of shared masses between the outlier isolates belonging to clades II, III, and IV might be responsible for these isolates’ low discrimination results. Additionally, we hypothesize that performing the analysis while including a higher number of non-clade I *C. auris* isolates should enhance the prediction ability of the CLADE-FINDER classifier.

One possible limitation to this study consists of the low number of available isolates belonging to *C. auris* clade V. Due to this limitation, it was not possible to include them in the model; as such, these isolates were used in the validation step as outgroup control, showing a distinct separation in the scatter plot from those belonging to the remaining clades (I-IV). This finding moves towards an optimization of the algorithm and further supports the future use of CLADE-FINDER for *C. auris* clades’ determination.

Additionally, sharing FTIR profiles among different centers is not yet a routine procedure due to the rigorous standardization of grow conditions (time, temperature, medium), which implies the creation of in-house databases or training models; further steps will be necessary in the future to implement this possibility and share pathogen spectra profiles.

Regarding the feasibility of the FTIR spectroscopic approach, it is worth considering that the whole process, starting from FTIR spectra generation, going through preprocessing, and ending with cluster typing, takes from 45 min to one hour depending on the number of samples analyzed. Moreover, this technique can be easily implemented in microbiology laboratories equipped with an FTIR spectrometer due to its speed, ease of performance, and low cost (about EUR 4 per sample).

If we compare FTIR’s technical and analytical features with the WGS approach, the first difference turns out to be that the former is a powerful strategy compared to the latter; once data are obtained, WGS analysis can be labor-intensive because of the absence of standardized bioinformatics tools and inadequate comparative genome databases, especially for fungal pathogens. Moreover, a WGS technical procedure requires specialized personnel and higher costs.

In conclusion, the CLADE-FINDER model for *C. auris* has shown great promise in accurately identifying different clades with high accuracy and reliability. The integration of FTIR spectroscopy and advanced machine learning techniques offers a rapid and cost-effective alternative to traditional methods, making it particularly valuable in resource-limited settings. While the model performed well overall, the presence of uncertain classifications and overlaps between clades indicate areas for further improvement. Addressing these issues through additional data collection, the refinement of spectral features, and the enhancement of the classification algorithms will further enhance the model’s accuracy and robustness, ultimately aiding in the effective epidemiological monitoring and management of *C. auris* [22,23,24].

## Figures and Tables

**Table 1:** Confusion matrix for the model training spectra, showing the clade classification and accuracy percentages of the IR Biotyper system for *Candida auris* clades I, II, III, and IV.

	CLADE I	CLADE II	CLADE III	CLADE IV	Class recall
CLADE I	**54**	-	-	-	100%
CLADE II	-	**34**	-	-	100%
CLADE III	-	-	**49**	-	100%
CLADE IV	-	-	-	**70**	100%
Class precision	100%	100%	100%	100%	

**Table 2:** Result overview of *Candida auris* IR Biotyper AI clade classification.

Isolate ID	Origin	Clade	Tot AVE n. ^a^	Best Result (AVE n.)	2nd Best Result (AVE n.)	Iso. Score ^b^
GE1	Italy	I	7	Clade I (5)	Clade III (2)	67.2
GE2	Italy	I	7	Clade I (5)	Clade III (2)	69.1
GE3	Italy	I	7	Clade I (5)	Clade III (2)	65.3
GE4	Italy	I	5	Clade I (4)	Clade III (1)	77.0
GE5	Italy	I	5	Clade I (4)	Clade III (1)	72.5
GE6	Italy	I	6	Clade I (5)	Clade III (1)	78.5
GE7	Italy	I	8	Clade I (6)	Clade III (2)	67.2
GE8	Italy	I	6	Clade I (5)	Clade III (1)	83.3
GE9	Italy	I	7	Clade I (5)	Clade III (2)	67.9
GE10	Italy	I	5	Clade I (4)	Clade III (1)	74.6
GE11	Italy	I	5	Clade I (4)	Clade III (1)	80.0
GE12	Italy	I	5	Clade I (4)	Clade III (1)	80.0
GE13	Italy	I	4	Clade I (3)	Clade III (1)	75.0
GE14	Italy	I	5	Clade I (4)	Clade III (1)	69.2
GE15	Italy	I	3	Clade I (2)	Clade III (1)	55.4
GE16	Italy	I	5	Clade I (4)	Clade III (1)	77.3
GE17	Italy	I	4	Clade I (3)	Clade III (1)	69.2
GE18	Italy	I	5	Clade I (4)	Clade III (1)	79.4
GE19	Italy	I	5	Clade I (4)	Clade III (1)	77.4
GE20	Italy	I	5	Clade I (4)	Clade III (1)	78.0
GE21	Italy	I	4	Clade I (3)	Clade III (1)	13.6
GE23	Italy	I	4	Clade I (3)	Clade III (1)	75.0
GE24	Italy	I	4	Clade I (3)	Clade III (1)	75.0
GE25	Italy	I	3	Clade I (2)	Clade III (1)	56.9
GE26	Italy	I	4	Clade I (3)	Clade III (1)	71.0
GE28	Italy	I	4	Clade I (3)	Clade III (1)	75.0
GE29	Italy	I	4	Clade I (3)	Clade III (1)	5.7
GE30	Italy	I	4	Clade I (3)	Clade III (1)	67.5
GE31	Italy	I	5	Clade I (4)	Clade III (1)	77.1
GE32	Italy	I	5	Clade I (4)	Clade III (1)	66.5
GE33	Italy	I	5	Clade I (4)	Clade III (1)	76.1
GE35	Italy	I	5	Clade I (4)	Clade III (1)	76.0
GE36	Italy	I	5	Clade I (4)	Clade III (1)	77.0
GE37	Italy	I	5	Clade I (4)	Clade III (1)	77.1
GE38	Italy	I	5	Clade I (4)	Clade III (1)	74.5
GE39	Italy	I	4	Clade I (3)	Clade III (1)	69.1
GE40	Italy	I	5	Clade I (4)	Clade III (1)	73.4
GE41	Italy	I	5	Clade I (4)	Clade III (1)	68.9
GE42	Italy	I	5	Clade I (4)	Clade III (1)	71.6
GE43	Italy	I	5	Clade I (4)	Clade III (1)	76.2
B11098	Pakistan	I	4	Clade IV (3)	Clade I (1)	9.2
B11205	India	I	2	Clade I (1)	Clade IV (1)	0.0
B8441	Pakistan	I	3	Clade I (3)		100.0
03-10-62	South Korea	II	4	Clade II (3)	Clade IV (1)	46.9
03-10-63	South Korea	II	4	Clade II (4)		97.2
03-10-65	Japan	II	3	Clade I (3)		37.6
05-15-51	South Africa	III	4	Clade I (3)	Clade IV (1)	49.5
08-11-93	Spain	III	4	Clade IV (4)		10.2
08-11-96	Spain	III	4	Clade III (4)		83.6
129628	Brasil	IV	2	Clade IV (2)		100.0
MOL-448		IV	2	Clade IV (2)		100.0
MOL-353	South Africa	IV	3	Clade IV (3)		100.0
11-14-06	Colombia	IV	4	Clade IV (4)		100.0
05-15-21	Venezuela	IV	4	Clade IV (4)		75.0
09-01-05	Venezuela	IV	3	Clade IV (3)		100.0
11-10-18	Iran	V	8	Clade IV (8)		100.0
13-10-56	Iran	V	8	Clade IV (8)		52.8
13-10-90	Iran	V	8	Clade II (6)	Clade I (2)	46.7

^a^ Total number of average FTIR spectra analyzed per isolate. ^b^ High reliability, indicated in green; moderate reliability, marked in yellow; and no reliability, represented in red, reported for isolate classification scores of 80 or higher; 20 or higher, but lower than 80; and lower than 20, respectively.

## Data Availability

The original contributions presented in the study are included in the article, further inquiries can be directed to the corresponding authors.
